# Use of activity tracking in major visceral surgery—the Enhanced Perioperative Mobilization (EPM) trial: study protocol for a randomized controlled trial

**DOI:** 10.1186/s13063-017-1782-1

**Published:** 2017-02-21

**Authors:** Steffen Wolk, Theresa Meißner, Sebastian Linke, Benjamin Müssle, Ann Wierick, Andreas Bogner, Dorothée Sturm, Nuh N. Rahbari, Marius Distler, Jürgen Weitz, Thilo Welsch

**Affiliations:** Department of Visceral, Thoracic and Vascular Surgery, University Hospital Carl Gustav Carus, Technical University of Dresden, Fetscherstrasse 74, 01307 Dresden, Germany

**Keywords:** Activity tracking, Postoperative recovery, ERAS, Fast-track surgery, Randomized controlled trial, RCT

## Abstract

**Background:**

Enhanced recovery after surgery (ERAS) programs are aimed at minimizing postoperative stress and accelerating postoperative recovery by implementing multiple perioperative principles. “Early mobilization” is one such principle, but the quality of assessment and monitoring is poor, and evidence of improved outcome is lacking. Activity trackers allow precise monitoring and automatic feedback to the patients to enhance their motivation for early mobilization. The aim of the study is to monitor and increase the postoperative mobilization of patients by giving them continuous automatic feedback in the form of a step count using activity-tracking wristbands.

**Methods/design:**

Patients undergoing elective open and laparoscopic surgery of the colon, rectum, stomach, pancreas, and liver for any indication will be included. Further inclusion criteria are age between 18 and 75 years, American Society of Anesthesiologists Physical Status class less than IV, and a signed informed consent form. Patients will be stratified into two subgroups, laparoscopic and open surgery, and will be randomized 1:1 for automatic feedback of their step count using an activity tracker wristband. The control group will have no automatic feedback. The sample size (*n* = 30 patients in each of the four groups, overall *n* = 120) is calculated on the basis of an assumed difference in step count of 250 steps daily (intervention group versus control group). The primary study endpoint is the average step count during the first 5 postoperative days; secondary endpoints are the percentage of patients in the two groups who master the predefined mobilization (step count) targets, assessment of additional activity data obtained from the devices, assessment of preoperative mobility, length of hospital and intensive care unit stays, number of patients who receive physiotherapy, 30-day mortality, and overall 30-day morbidity.

**Discussion:**

Early mobilization is a key element of ERAS. However, enhanced early mobilization is difficult to define, to assess objectively, and to implement in clinical practice. Consequently, there is a discrepancy between ERAS targets and actual practice, especially in patients undergoing major visceral surgery. This study is the first randomized controlled trial investigating the use and feasibility of activity tracking to monitor and enhance postoperative early mobilization.

**Trial registration:**

ClinicalTrials.gov identifier: NCT02834338. Registered on 15 June 2016.

**Electronic supplementary material:**

The online version of this article (doi:10.1186/s13063-017-1782-1) contains supplementary material, which is available to authorized users.

## Background

“Fast-track surgery”, or “enhanced recovery after surgery” (ERAS), is a clinical pathway for a variety of surgical procedures. The aim of ERAS programs is to minimize postoperative stress and accelerate postoperative recovery through the implementation of multiple pre-, intra-, and postoperative ERAS elements (e.g., preoperative fasting and bowel preparation or antimicrobial prophylaxis, intraoperative analgesia and fluid management, postoperative pain and drainage management, nutritional care, early mobilization) [1–3]. Nowadays, ERAS is considered standard for the perioperative management of colorectal surgical patients in many centers [4–6]. ERAS protocols have resulted in significantly shorter hospital stays and overall complications [5]. The advantages of ERAS based on the available data seem to outweigh possible disadvantages, although the quality of the data does not justify a final implementation as standard care. Nevertheless, ERAS has produced attractive results and consequently has been used in gastrointestinal (GI), hepatopancreatic, or esophageal surgery with similar results [7–12]. In recent years, ERAS programs have also been adapted to major visceral surgery of the upper GI tract [7–11]. A majority of the ERAS elements are well defined, whereas there is a lack of evidence for early mobilization protocols and monitoring. Studies on early mobilization follow different protocols and definitions. For example, Anderson et al. and Gatt et al. recommended that patients get out of bed on the day of the operation and mobilize on the floor on the first postoperative day (POD) [13, 14]. Interestingly, Anderson et al. included only patients undergoing left or right colectomy, whereas Gatt et al. included patients who underwent all kinds of colorectal operations without consideration of the severity of surgery [13, 14]. Wind et al. defined enhanced mobilization as getting out of bed more than 2 h on the day of the operation up to 8 h at the second POD after laparoscopic or open colorectal surgery [15, 16]. The findings of ERAS programs on recovery of surgical patients were subsequently transferred to ERAS programs for liver and pancreatic surgery [7, 11, 16–18]. Drawbacks of these studies are that “early mobilization” was not defined consistently and that no distinction was made between standard and early mobilization. That is why there is no solid evidence that early mobilization in the ERAS setting is beneficial. A recent study done at our center emphasizes the fact that almost 50% of our patients after surgery of the upper GI tract do not request enhanced mobilization, indicating that ERAS principles and the mobilization targets of the available studies cited above were not achieved [19].

Consequently, novel or innovative methods have to be evaluated to provide objective and precise monitoring of patient mobilization, as well as to increase the intrinsic motivation for early mobilization after major visceral surgery. One possibility could be the use of activity trackers that allow automatic feedback to patients to enhance their motivation for early mobilization. Technological progress has fostered the development of wearable devices for “self-tracking,” including the tracking of physical activity [20]. The first cohort study with 150 participants investigating the effect of a modern wearable activity trackers upon postsurgical mobility recovery during hospitalization found a significant relationship between the early recovery step count and length of hospital stay in elderly cardiac surgery patients [21]. There are no reports of randomized controlled trials (RCTs) involving activity tracking of postoperative in-hospital mobilization. The aim of the present study is to evaluate the effect of an automatic feedback system using activity trackers versus no feedback on postoperative step count and recovery after major visceral surgery.

## Methods/design

### Trial design

The Enhanced Perioperative Mobilization (EPM) trial is a randomized, controlled, single-center trial comparing the effect of automatic feedback of postoperative physical activity using activity tracker wristbands after laparoscopic and open major visceral surgery. The trial design is in accordance with the Standard Protocol Items: Recommendations for Interventional Trials (SPIRIT) statement (see Additional file 1).

### Study population

The study population consists of all patients scheduled for elective laparoscopic and open surgery of the colon and rectum (colectomy, hemicolectomy, segment resection, rectum extirpation, deep anterior rectum resection, sigmoid resection, proctocolectomy), of the stomach (total, subtotal, and atypical gastric resections), of the pancreas (any kind of pancreatic resection), and of the liver (hemihepatectomy, atypical resection, anatomical segment resection). Further inclusion criteria are age equal to or older than 18 years up to 75 years, American Society of Anesthesiologists Physical Status class less than IV, and a completed informed consent form. Exclusion criteria are emergency surgery, mental inability to complete postoperative assessment protocols, or preoperatively immobile patients. Dropout from the study is allowed in cases of nonresectability, postoperative mechanical ventilation >12 h, prolonged stay in the intensive care unit (ICU) >48 h, lack of compliance with wearing the activity tracker wrist band, or allergic reactions to the wrist band. All reasons for dropouts will be analyzed, and these patients will be followed.

The EPM trial is to be conducted in line with either the Declaration of Helsinki or the laws and regulations of Germany, whichever provides the greatest protection to the patients. The study protocol was approved by the local ethical committee of Technical University Dresden (decision number EK226062016). This study is registered with ClinicalTrials.gov under the unique identifying number NCT02834338.

### Randomization and procedures

Patients are to be screened for eligibility on the day of admission. After participants give their informed consent to participate in the study, the International Physical Activity Questionnaire (IPAQ) will be administered to assess the preoperative mobilization, and the patients will receive preoperative counseling before surgery.

To avoid bias due to differences between laparoscopic and open surgery, two arms will be built before randomization: a laparoscopic arm and an open surgery arm. The study population will be stratified by surgery type (open and laparoscopic surgery). The randomization (control group versus intervention group) will be performed intraoperatively after the surgeon has confirmed the resectability using a block randomization with fixed block sizes in a 1:1 allocation ratio. The randomization sequences will be generated using the R statistical software package (R version 3.1.3, R Foundation for Statistical Computing, Vienna, Austria). Block size will be kept confidential until completion of recruitment.

The wristband will be worn continuously after the operation to count the patient’s steps 24 h per day until the beginning of POD 6. The Polar Loop activity tracker with FlowSync and Polar Flow software will be used for activity tracking (Polar Electro GmbH, Büttelborn, Germany). The device has been approved by the Medical Devices Act (EU Medical Device Directive 93/42/EWG/CD 0537) and is recommended for medical use.

**Fig. 1:**
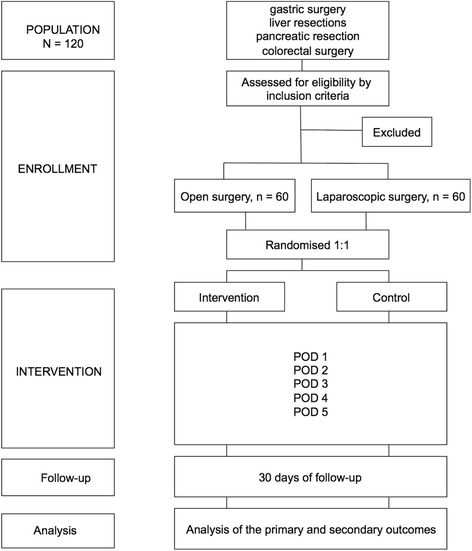
Flowchart. *EPM* Enhanced Perioperative Mobilization trial, *POD* Postoperative day

The control group will wear an activity tracker wristband with a display covered with adhesive tape so that the step count cannot be read. The study nurse will verify the covered display a few times daily. The intervention group will receive an unblinded wristband. The handling of the activity trackers will be explained to the patients, and a predefined mobilization endpoint (step count) for the first 5 PODs will be targeted. The target step count was set at the 85% quartile on the basis of results a previous pilot study (Table 1). Patients older than 75 years of age were excluded from the analysis in the pilot study. The median age of this group was 60 years (IQR 52.5–70.5 years). A major pancreatic resection was performed in 35% of the patients, a major liver resection in 30%, a colorectal resection in 25%, and a gastric resection in 10%. A surgical fellow or a study nurse will assess and monitor the patient twice daily, between 9:00 a.m. and 11:00 a.m. and between 3:00 p.m. and 5:00 p.m., throughout the patient’s hospital stay to read the step count, to ensure its proper use and functioning, and to communicate the automatic feedback results. The study flowchart is shown in Fig. 1.

**Table 1:** Mobilization targets in steps per day

	POD 1	POD 2	POD 3	POD 4	POD 5
Open surgery arm (*n* = 20)	500	620	800	1400	1400
Laparoscopic arm (*n* = 5)	1900	2300	2900	3400	3400

*POD* Postoperative day

Data are presented as 85% quartile based on *n* = 25 patients (results of a pilot study)

### Study aim and objectives

The aim of the study is to increase the postoperative mobilization of the patients by giving them continuous feedback on their step count using activity-tracking wristbands. The primary endpoint of this study is the average step count in the first five POD in the intervention and control groups. Secondary endpoints are the percentage of patients in the two groups who master the predefined mobilization (i.e., step-count) targets, assessment of the activity data obtained from the devices (distance, activity time, inactivity, calorie consumption, compliance), assessment of preoperative mobility with the IPAQ [22], length of hospital and ICU stays (from the first POD until day of discharge), number of patients who receive physiotherapy, 30-day mortality, and 30-day overall morbidity according the Clavien-Dindo classification [23].

### Statistical considerations and sample size calculation

The sample size estimation was based on our results in a pilot study with 30 participants, 25 of whom were analyzed to collect data about standard mobilization on the first 5 PODs following major visceral surgery. Based on those data, an increase in the daily step count by 250 steps/day with an SD of 290 steps/day was estimated. To achieve 80% power with a two-sided *P* value <0.05 and a dropout rate of 12%, the total sample size calculated with a two-tailed, unpaired *t* test was 120 patients with 30 patients in each group. 

The statistical analysis will be done on an intention-to-treat basis and will be performed with Statistical Package for Social Science software version 18.0 (SPSS, Chicago, IL, USA). The Mann-Whitney *U* test will be used to compare continuous variables, and Fisher’s exact test will be used for categorical variables. Two-sided *P* values will always be computed, and a difference will be considered statistically significant at *P* ≤ 0.05. The primary outcome is the average step count over the first 5 PODs of the patients in the intervention and control goups, separated into the laparoscopic and open surgery arms, respectively. Statistical testing of the outcome parameter will be performed using a regression analysis of repeated measures. In addition, the increase of mobilization within the groups will be analyzed using the repeated measures ANOVA. Logistic regression analyses will be computed to identify factors determining the patient cohort that achieved the mobilization targets (secondary endpoint). The following variables will be considered for the regression analysis: age, sex, body mass index (BMI), operative time, oncological versus nononcological indications, type of surgery (pancreatic, liver, intestinal, gastric), preoperative IPAQ score, and technique of surgery (laparoscopic versus open). Factors that are significant in univariate analysis will be further considered for a multivariate regression analysis.

### Study implementation

**Fig. 2:**
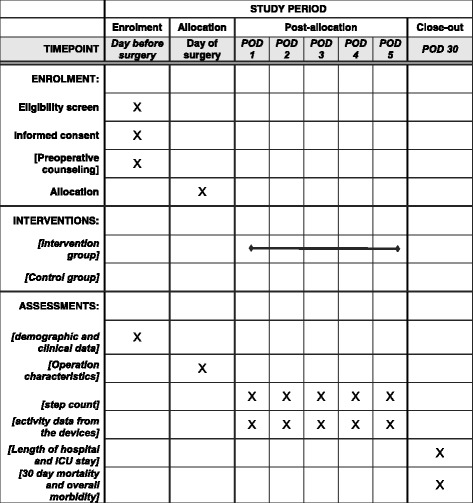
Study implementation of the Enhanced Perioperative Mobilization trial. *ICU* Intensive care unit, *POD* Postoperative day

Patients will be assessed daily by a surgical fellow or a study nurse throughout their hospital stay. The whole team, including nurses and physiotherapists, is trained for the mobilization with the study patients and assists the patients on the arm without the activity-tracking device to avoid bias. The data obtained from the activity tracker will be synchronized automatically at POD 6 according to the user manual. The team will also follow the patients after their discharge and perform all data collection in an attempt to minimize observer bias. All outcome parameters will be recorded before the operation and on PODs 1, 2, 3, 4, and 5. At a follow-up visit on POD 30, data on all endpoints and patient characteristics will be gathered. Figure 2 shows the study implementation in accordance with the SPIRIT statement.

### Documentation and data handling

All protocol-required information collected during the trial will be entered into the case report forms (CRFs). The completed CRFs will be reviewed, signed, and analyzed by the investigator or by a designated subinvestigator. During the trial, patients will be identified solely by means of their year of birth and individual identification code (screening number, randomization number). Trial findings will be stored in accordance with local data protection law and International Council for Harmonisation good clinical practice guidelines, and they will be handled in the strictest confidence. For the protection of these data, organizational procedures will be implemented to prevent the distribution of data to unauthorized people.

## Discussion

To our knowledge, the EPM trial is the first RCT to compare the effect of wearable activity tracker devices to enhance patients’ motivation for early mobilization in the ERAS setting. Cook et al. completed the first cohort study using a mobility tracker with 150 patients after cardiac surgery. The study showed that there was a significant relationship between the number of steps taken in the early recovery period, length of stay, and dismissal disposition [21]. However, in this setting, only the relationship between step count and length of hospital stay was investigated, and there was no automatic feedback to the patients. Our present RCT will be the first study addressing surgical patients with the aim of enhancing patients’ intrinsic motivation for more postoperative mobilization through an automatic feedback mechanism. Martin et al. showed in an RCT that an automated mobile health intervention with a tracking device and a texting component can increase a patient’s physical activity, and it was successfully used as a behavior change driver in ambulatory cardiac patients [24]. A problem was that only the text-messaging component of that study increased the physical activity. Chan et al. used pedometers to increased physical activity in a sedentary population of 106 participants. They suggested a count of 10,000 steps per day as an effective target for prevention of disease and promotion of a healthier lifestyle [25]. Bed rest is associated with several complications, such as pneumonia, but solid data for visceral surgery are lacking [26, 27]. However, self-tracking can be used in chronic disease cases as well. Authors of a recent meta-analysis of activity monitor-based counseling studies concluded that there are beneficial effects derived from self-tracking on physical activity, blood glucose, systolic blood pressure, and BMI in patients with diabetes [28]. In patients with chronic obstructive pulmonary disease and chronic heart failure, the benefit was unclear owing to limited or nonexistent data [28]. There is still a lack of evidence in other chronic diseases [29]. The problem with early mobilization of surgical patients is the imposed immobility by devices such as drains and catheters [19]. Even in the ERAS setting, an increase of early mobilization is difficult to implement [30]. For example only 20–28% of patients were mobilized on the first POD after liver surgery, despite targets such as “four times daily” [11, 31, 32]. Our first pilot study indicated a discrepancy between ERAS targets and actual practice in patients with comorbidities undergoing major visceral surgery. The data underlined the need for a prompt redefinition of ERAS mobilization targets [19]. The use of activity trackers can be useful when defining these mobilization targets, monitoring postoperative patient parameters, and helping to implement the ERAS principles by increasing the patient’s intrinsic motivation.

The present trial includes many types of operations, including hepatopancreatic, GI, and colorectal surgery, which might have a different impact on the patients’ postoperative physiology and ability to ambulate. This broad range of operations was intended to increase the applicability and generalizability of the study. As a result, minor effects of the activity-tracking feedback that might be confined to only one type of surgery might be missed. However, our pilot study, although not powered for this endpoint, showed no significant difference in the achieved postoperative step counts between the different operations.

The present study further includes patients within a broad age range and associated fitness level. Differences in functional abilities naturally occurring with aging [33] and that might be aggravated after surgery can further bias the study, but they might contribute to the applicability of the results. In addition, the potential for discrepancies between the self-reported preoperative mobility assessed by the IPAQ and actual quantitative mobility cannot be excluded. In addition, the IPAQ was not designed for patients in a hospital setting (i.e., inpatients). Nevertheless, the IPAQ is considered an appropriate tool to assess physical activity in daily life, and results derived from it will be analyzed as a secondary endpoint in the present trial [34].

## Trial status

Participants are currently being recruited. The first patient was enrolled in July 2016.

## Additional file

Additional file 1: SPIRIT checklist: description of the trial design in accordance with the SPIRIT statement. (DOC 120 kb)

## Abbreviations

BMI: Body mass index
CRF: Case report form
EPM: Enhanced Perioperative Mobilization
ERAS: Enhanced recovery after surgery
GI: Gastrointestinal
ICU: Intensive care unit
IPAQ: International Physical Activity Questionnaire
POD: Postoperative day
RCT: Randomized controlled trial
SPIRIT: Standard Protocol Items: Recommendations for Interventional Trials

## References

[1] Gustafsson UO, Scott MJ, Schwenk W, Demartines N, Roulin D, Francis N, McNaught CE, MacFie J, Liberman AS, Soop M (2012). Guidelines for perioperative care in elective colonic surgery: Enhanced Recovery After Surgery (ERAS®) Society recommendations. Clin Nutr.

[2] Lassen K, Soop M, Nygren J, Cox PB, Hendry PO, Spies C, von Meyenfeldt MF, Fearon KC, Revhaug A, Norderval S (2009). Consensus review of optimal perioperative care in colorectal surgery: Enhanced Recovery After Surgery (ERAS) Group recommendations. Arch Surg.

[3] Nygren J, Thacker J, Carli F, Fearon KC, Norderval S, Lobo DN, Ljungqvist O, Soop M, Ramirez J, Enhanced Recovery After Surgery Society (2012). Guidelines for perioperative care in elective rectal/pelvic surgery: Enhanced Recovery After Surgery (ERAS®) Society recommendations. Clin Nutr.

[4] Kehlet H (2011). Fast-track surgery—an update on physiological care principles to enhance recovery. Langenbecks Arch Surg.

[5] Spanjersberg WR, Reurings J, Keus F, van Laarhoven CJ (2011). Fast track surgery versus conventional recovery strategies for colorectal surgery. Cochrane Database Syst Rev.

[6] Muller S, Zalunardo MP, Hubner M, Clavien PA, Demartines N, Zurich Fast Track Study Group (2009). A fast-track program reduces complications and length of hospital stay after open colonic surgery. Gastroenterology.

[7] Balzano G, Zerbi A, Braga M, Rocchetti S, Beneduce AA, Di Carlo V (2008). Fast-track recovery programme after pancreatico-duodenectomy reduces delayed gastric emptying. Br J Surg.

[8] Coolsen MM, Wong-Lun-Hing EM, van Dam RM, van der Wilt AA, Slim K, Lassen K, Dejong CH (2013). A systematic review of outcomes in patients undergoing liver surgery in an enhanced recovery after surgery pathways. HPB (Oxford).

[9] Ford SJ, Adams D, Dudnikov S, Peyser P, Rahamim J, Wheatley TJ, Berrisford RG, Sanders G (2014). The implementation and effectiveness of an enhanced recovery programme after oesophago-gastrectomy: a prospective cohort study. Int J Surg.

[10] Tang J, Humes DJ, Gemmil E, Welch NT, Parsons SL, Catton JA (2013). Reduction in length of stay for patients undergoing oesophageal and gastric resections with implementation of enhanced recovery packages. Ann R Coll Surg Engl.

[11] van Dam RM, Hendry PO, Coolsen MM, Bemelmans MH, Lassen K, Revhaug A, Fearon KC, Garden OJ, Dejong CH, Enhanced Recovery After Surgery (ERAS) Group (2008). Initial experience with a multimodal enhanced recovery programme in patients undergoing liver resection. Br J Surg.

[12] Shewale JB, Correa AM, Baker CM, Villafane-Ferriol N, Hofstetter WL, Jordan VS, Kehlet H, Lewis KM, Mehran RJ, Summers BL (2015). Impact of a fast-track esophagectomy protocol on esophageal cancer patient outcomes and hospital charges. Ann Surg.

[13] Anderson AD, McNaught CE, MacFie J, Tring I, Barker P, Mitchell CJ (2003). Randomized clinical trial of multimodal optimization and standard perioperative surgical care. Br J Surg.

[14] Gatt M, Anderson AD, Reddy BS, Hayward-Sampson P, Tring IC, MacFie J (2005). Randomized clinical trial of multimodal optimization of surgical care in patients undergoing major colonic resection. Br J Surg.

[15] Ramírez JM, Blasco JA, Roig JV, Maeso-Martínez S, Casal JE, Esteban F, Lic DC, Spanish working group on fast track surgery (2011). Enhanced recovery in colorectal surgery: a multicentre study. BMC Surg.

[16] Wind J, Hofland J, Preckel B, Hollmann MW, Bossuyt PM, Gouma DJ, van Berge Henegouwen MI, Fuhring JW, Dejong CH, van Dam RM (2006). Perioperative strategy in colonic surgery; LAparoscopy and/or FAst track multimodal management versus standard care (LAFA trial). BMC Surg.

[17] Stoot JH, van Dam RM, Busch OR, van Hillegersberg R, De Boer M, Olde Damink SW, Bemelmans MH, Dejong CH, Enhanced Recovery After Surgery (ERAS) Group (2009). The effect of a multimodal fast-track programme on outcomes in laparoscopic liver surgery: a multicentre pilot study. HPB (Oxford).

[18] Ni CY, Yang Y, Chang YQ, Cai H, Xu B, Yang F, Lau WY, Wang ZH, Zhou WP (2013). Fast-track surgery improves postoperative recovery in patients undergoing partial hepatectomy for primary liver cancer: a prospective randomized controlled trial. Eur J Surg Oncol.

[19] Wolk S, Distler M, Mussle B, Sothje S, Weitz J, Welsch T (2016). Adherence to ERAS elements in major visceral surgery—an observational pilot study. Langenbecks Arch Surg.

[20] Fox S, Duggan M (2013). Tracking for health.

[21] Cook DJ, Thompson JE, Prinsen SK, Dearani JA, Deschamps C (2013). Functional recovery in the elderly after major surgery: assessment of mobility recovery using wireless technology. Ann Thorac Surg.

[22] Scholes S, Bridges S, Ng Fat L, Mindell JS (2016). Comparison of the Physical Activity and Sedentary Behaviour Assessment Questionnaire and the Short-Form International Physical Activity Questionnaire: an analysis of Health Survey for England data. PLoS One.

[23] Dindo D, Demartines N, Clavien PA (2004). Classification of surgical complications: a new proposal with evaluation in a cohort of 6336 patients and results of a survey. Ann Surg.

[24] Martin SS, Feldman DI, Blumenthal RS, Jones SR, Post WS, McKibben RA, Michos ED, Ndumele CE, Ratchford EV, Coresh J (2015). mActive: a randomized clinical trial of an automated mHealth intervention for physical activity promotion. J Am Heart Assoc.

[25] Chan CB, Ryan DA, Tudor-Locke C (2004). Health benefits of a pedometer-based physical activity intervention in sedentary workers. Prev Med.

[26] Convertino VA (1997). Cardiovascular consequences of bed rest: effect on maximal oxygen uptake. Med Sci Sports Exerc.

[27] Kehlet H, Wilmore DW (2002). Multimodal strategies to improve surgical outcome. Am J Surg.

[28] Vaes AW, Cheung A, Atakhorrami M, Groenen MT, Amft O, Franssen FM, Wouters EF, Spruit MA (2013). Effect of ‘activity monitor-based’ counseling on physical activity and health-related outcomes in patients with chronic diseases: a systematic review and meta-analysis. Ann Med.

[29] Allet L, Knols RH, Shirato K, de Bruin ED (2010). Wearable systems for monitoring mobility-related activities in chronic disease: a systematic review. Sensors.

[30] Gustafsson UO, Hausel J, Thorell A, Ljungqvist O, Soop M, Nygren J, Enhanced Recovery After Surgery Study Group (2011). Adherence to the enhanced recovery after surgery protocol and outcomes after colorectal cancer surgery. Arch Surg.

[31] Hendry PO, van Dam RM, Bukkems SF, McKeown DW, Parks RW, Preston T, Dejong CH, Garden OJ, Fearon KC, Enhanced Recovery After Surgery (ERAS) Group (2010). Randomized clinical trial of laxatives and oral nutritional supplements within an enhanced recovery after surgery protocol following liver resection. Br J Surg.

[32] Koea JB, Young Y, Gunn K (2009). Fast track liver resection: the effect of a comprehensive care package and analgesia with single dose intrathecal morphine with gabapentin or continuous epidural analgesia. HPB Surg.

[33] Sallis JF (2000). Age-related decline in physical activity: a synthesis of human and animal studies. Med Sci Sports Exerc.

[34] Steene-Johannessen J, Anderssen SA, van der Ploeg HP, Hendriksen IJ, Donnelly AE, Brage S, Ekelund U (2016). Are self-report measures able to define individuals as physically active or inactive?. Med Sci Sports Exerc.

